# Analysis of deprescription strategies of proton pump inhibitors in primary care: a narrative review

**DOI:** 10.1017/S1463423623000026

**Published:** 2023-02-15

**Authors:** Miguel Del-Pino, Emilio J. Sanz

**Affiliations:** 1 Servicio Canario de la Salud, Tenerife, Spain. Canary Islands; 2 Facultad de Ciencias de la Salud. Universidad de La Laguna, La Laguna, Tenerife, Spain; 3 Complejo Hospital Universitario de Canarias, Tenerife, Spain

## Abstract

**Background:**

Since the introduction of omeprazole in 1989, proton pump inhibitors (PPIs) have become the mainstream of treatment for acid-related pathologies, but nowadays, it is estimated that between 20% and 80% of people worldwide who are using PPIs are doing so without an approved indication. Overusing PPIs is known to involve a tremendous cost in financial terms, and many western countries have reported high spending on these medicines.

**Objective:**

We conducted a narrative review to evaluate PPI deprescription strategies carried out entirely or in collaboration with primary care and to identify factors that could influence the success of these strategies.

**Method:**

This review was conducted in November 2022, following PRISMA guidelines. Four databases were searched: PubMed, Web of Science, Scopus and CINAHL Complete, using the MeSH terms ‘proton pump inhibitors’ AND ‘deprescriptions’.

**Results:**

The search with the established criteria found eight studies. The different success rates obtained by the various studies analysed in this review may be due to the different methodologies used when establishing the protocols, sample selection and monitoring of the results.

**Conclusion:**

We can conclude that the two factors related to the most successful strategies were a) the clarity and simplicity of the de-escalation protocols, in which patients were instructed on the measures to follow in the event of the reappearance of symptoms, and b) the training of the physicians responsible for deprescribing. Long-term conclusions cannot be drawn about the effectiveness of these protocols, given that the studies are limited in time. Other barriers to generalizing the results are the small sample size and the absence of control groups.

## Introduction

Since the introduction of omeprazole in 1989, proton pump inhibitors (PPIs) have become the mainstream treatment for acid-related pathologies (Strand *et al*., 2017; Lanas-Gimeno *et al*., 2019). Compared with previous drugs, such as synthetic prostaglandin analogues, anticholinergics and histamine receptor antagonists (antiH_2_), they are very well tolerated by patients. They have an excellent safety profile and a superior acid suppression capacity than antiH_2_ (Strand *et al*., 2017).

The indications authorized for PPIs in Spain, as in many other countries, are as follows: gastroesophageal reflux disease (GERD), gastroduodenal ulcer, *Helicobacter Pylor*i eradication treatment, Zollinger-Ellison syndrome and the prevention of gastropathy due to chronic use of NSAIDs in patients at risk of bleeding (older than 65 years, patients with a previous uncomplicated gastroduodenal ulcer, and the concomitant use of acetylsalicylic acid, corticosteroids or oral anticoagulants) (AEMPS, 2021).

### Amount of use

It is estimated that between 20% and 80% of people worldwide who are using PPIs are doing so without an approved indication (Lanas-Gimeno *et al*., 2019) (Savarino *et al.*, 2018) (Boghossian *et al.*, 2017) (Farrell *et al.*, 2017) (Walsh *et al.*, 2016) (Lassalle *et al.*, 2020). Overuse of PPIs is known to involve a tremendous cost in financial terms.

Many Western countries have reported high spending on PPIs. As reflected in the Canadian Health Network (2013) report (Boghossian *et al.*, 2017), eight brands of PPIs were among the 100 highest-cost drugs, with esomeprazole ranked seventh on the list. According to that report, of the 7.8 trillion Canadian dollars of public spending invested in medicines in 2013, 249.6 million were allocated to PPIs (3.2% of total medicines cost for PPI). In France, PPI sales increased by around 20% between 2010 and 2013, with 80 million packages sold in 2013, placing esomeprazole, omeprazole and pantoprazole among the 30 best-selling drugs in pharmacies. In 2015, almost 30 % of the French adult population (more than 15,000,000 inhabitants over 18 years of age) consumed at least one package of PPIs. Almost half of them were new users of these drugs, and their indication was not documented in nearly a third of the cases (Lassalle *et al.*, 2020).

In England, in 2006, out of a total expenditure of 7 billion pounds, 425 million corresponded to PPIs (6.1%) (Boghossian *et al.*, 2017). The use of these drugs represented, in 2009, an expenditure of 13.6 billion US dollars throughout the world (Savarino *et al.*, 2018). It is the third most prescribed group of drugs in the United States (Reid *et al.*, 2012). Their cost is increased by using brand-name drugs instead of generics, with an estimated 5-year excess cost of using brand-name PPIs in the United States exceeding 47 billion US dollars (Graham and Tansel, 2018). In Spain, the pharmaceutical expenditure in 2018 was 10 927 million euros corresponding to 963 million packages invoiced. PPIs were the chemical subgroup with the highest consumption in the number of packages, with 65.5 million (Spanish Minister for Health, 2021). According to British data, a potential expenditure of 2 trillion pounds is invested unnecessarily annually in these drugs worldwide (Heidelbaugh *et al.*, 2012).

The use of PPI differs significantly from one country to another, whereas in some countries, these are ‘prescription-only drugs’ and in many others can be bought over the counter. For these reasons, some official statistics based on reimbursement could (severely) underestimate the actual consumption of these drugs.

It is worth mentioning that these high costs, and long-live prescriptions, are also accompanied by several relevant side effects. Some of the most relevant are abdominal discomfort and pain, constipation, diarrhoea, headache, insomnia, hyponatremia, osteoporosis, interstitial nephritis, B12 impaired absorption or *Campylobacter*, Salmonella or *C. diffcile* gastrointestinal infections. In some cases, ventricular arrhythmias associated with hypomagnesemia could occur.

### Inadequate indications

Many studies have analysed the use without indication of these drugs at the hospital and in primary care (Savarino *et al.*, 2018). In France, the misuse of PPIs has been documented to range from 40% to more than 80%, depending on the definition used (Lassalle *et al.*, 2020). In 2011, a study published in the United States (Reid *et al.*, 2012) developed a retrospective analysis of the suitability of the prescription of PPIs in patients discharged from different university hospitals in Colorado. The study concluded that 73% of almost one million patients received a PPI without adequate indication during hospitalization. Another study examined the initiation of PPI treatment in hospitalized patients unnecessarily and continued at discharge in western Pennsylvania (Thomas *et al.*, 2010): 70% of patients who had started a PPI and kept it at discharge did so inappropriately. The percentage of those who started it after admission to an intensive care unit (ICU) or a coronary unit was comparable to that of those who had been hospitalized outside these critical patient units, 68.7% vs 68.9%, respectively (*P* = 0.796). The study found that during the 4-year analysis period, the cost associated with inappropriate continuation of PPIs for 30 days after discharge was 3 million US dollars.

Gupta et al. (Gupta *et al.*, 2010) conducted a retrospective review of a randomized sample of patients admitted to the general medicine service of a Florida university hospital to determine the unnecessary continuation of discharge from PPIs initiated during admission in the period between August and October 2006. 73% of those admitted who began treatment with a PPI did so unnecessarily. Almost 70% (69%) of the patients who started an unnecessary treatment with a PPI maintained the same regimen at discharge. The most frequent causes of inappropriate prescription were stress ulcer prophylaxis in low-risk patients and gastrointestinal ulcer prophylaxis in patients taking only corticosteroids or anticoagulants with no other risk factor.

The inappropriate use of PPIs in primary care has also been widely studied. At this level of care, the continued use of PPIs after hospital discharge and the absence of a periodic review of patients who use these drugs on a chronic basis are the leading cause of inappropriate use (Savarino *et al.*, 2018). A study developed in 36 primary care centres in the Northeastern state of Mecklenburg-West Pomerania from Germany between 2006 and 2007 (Ahrens *et al.*, 2012), which analysed the prescription of PPIs recommended at discharge after hospital admission and its continuation in primary care, concluded that 52% of the cases in which a PPI was prescribed at discharge, there was no appropriate indication. Of these, 58% remained in primary care after one month and 42% after six months. According to that study, the most important factor associated with the appropriate vs inappropriate continuation of PPIs after discharge was the prescription of PPIs before hospitalization. Not to mention that two-thirds of inappropriate medication was started in the hospital.

Functional dyspepsia is another cause of PPI over-prescription, especially in the long term, since family doctors frequently indicate these drugs indefinitely without a periodic reassessment to establish the suitability of its continuation, the possibility of reducing their dose or even stopping them. The success of PPIs in these cases is low, ranging from 10% to 30% (Savarino *et al.*, 2018). According to a Cochrane systematic review published in 2017 (Pinto-Sanchez *et al.*, 2017), comparing PPI versus placebo, the number of patients that would need to be treated to get a benefit (NNT) is 11.

Gastroprotection with PPIs in patients under 65 years of age under treatment with NSAIDs without risk factors for gastrointestinal bleeding is another of the leading causes of poor indication of these drugs globally (Savarino *et al.*, 2018). A study published in the United States in 2002 (Laine *et al.*, 2002), carried out in 301 centres in 22 countries, compared the difference in risk of producing adverse effects in the upper gastrointestinal tract, such as bleeding, perforation or obstruction, among patients diagnosed with rheumatoid arthritis patients taking naproxen (NSAIDs) and those taking rofecoxib. The result was that the NNT to prevent one of these adverse effects with rofecoxib was 66 in those younger than 65, 25 in those older than 65 and 10 in those older than 75. The NNT to prevent an adverse effect in those without a history of previous gastrointestinal events was 51.

In 2016, a retrospective study in Tennessee (USA) (Ray *et al.*, 2016) analysed the hospital admission for bleeding from the upper gastrointestinal tract of patients receiving warfarin treatment. They differentiate between those who received concomitant therapy with PPI and those who had not received it. The investigators did not find a significant protective effect of concomitant PPI therapy in patients receiving warfarin who did not use antiplatelet drugs or NSAIDs.

The concomitant administration of oral anticoagulants such as low molecular weight heparins or warfarin with PPIs is also not indicated in patients without other risk factors for gastrointestinal bleeding since these drugs are not directly gastro-toxic (Savarino *et al.*, 2018). In the case of ticlopidine or clopidogrel administered in patients without risk factors, PPI treatment is not required, except that they are administered with <>ASA in the secondary prevention of myocardial ischaemia (Savarino *et al.*, 2018).

There are several approaches to the deprescription of PPI, both in in-patient care and in primary care. After a careful review of the available evidence published on deprescription strategies of PPI in primary care, a limited number of articles could be found. However, those represent the current state of knowledge on the efficacy and suitability of deprescription in primary care. All these studies and conclusions on the amount of use and the inadequate indications show a large room for improvement in the prescription of PPI, which should be reduced in almost all settings and places.

## Method

A review of studies on PPI deprescription strategies carried out entirely or in collaboration with primary care was carried out in November 2022, following PRISMA guidelines (Moher *et al.*, 2009). The search was done in English and Spanish, with no date limits. Four databases were searched: PubMed, Web of Science, Scopus and CINAHL Complete, using the MeSH terms ‘proton pump inhibitors’ AND ‘deprescriptions’. The two authors did the searches in parallel and contrasted the results. Studies that did not evaluate at least one PPI deprescription strategy were discarded, taking into account randomized, non-randomized intervention studies, systematic reviews and narrative reviews. Conferences or lectures, prescribing guides or deprescribing protocols that did not provide results were not evaluated.

As this study is a narrative review of published sources, no ethical assessment was deemed necessary.

**Figure 1.**
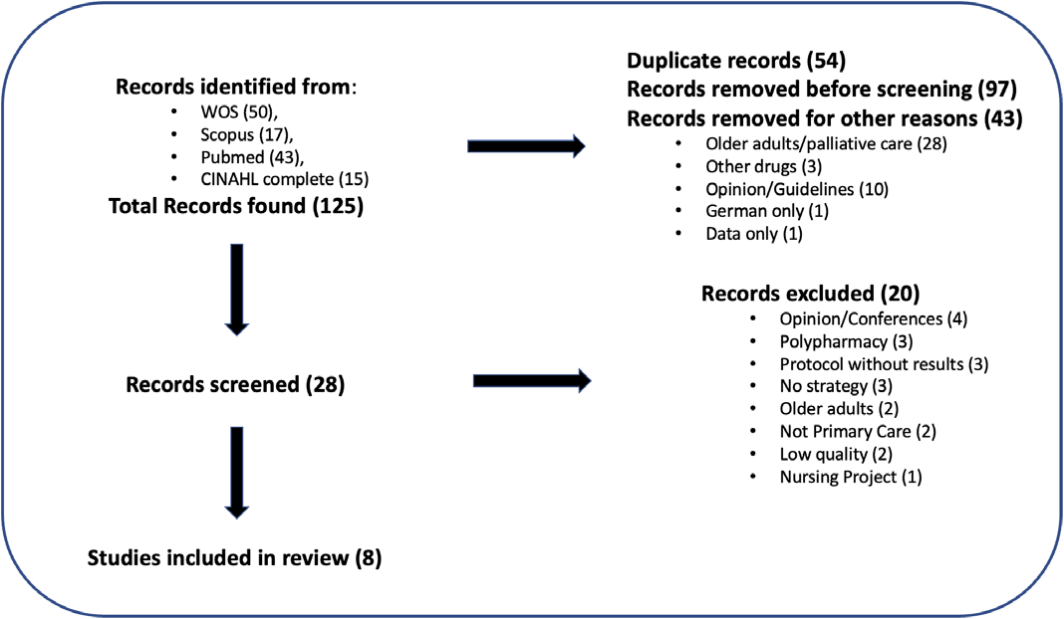
Bibliographic Search Strategy.

According to the PRISMA criteria (Moher *et al.*, 2009), a flow diagram of the searches is described in Figure 1.

## Results

The search with the established criteria found eight studies.

There are two systematic reviews, both published in 2017 and six studies published after them. The details of included studies are outlined in Table 1.

**Table 1.** Details of included studies

Study	Study design	Intervention	Results	Comment
Wilsdon *et al*., 2017 (20), Australia	Systematic review. 21 randomized and non-randomized studies carried out in Australia, New Zealand, North America, UK, France, Switzerland, Germany, the Netherlands and Israel. (Six studies were randomized, and of the remaining 15, two had a control group)	Deprescription of improperly prescribed PPIs ≥65 years, hospitalized patients, residents of community housing or nursing homes.	Deprescription interventions were effective in six studies. In the rest, they were inconclusive (11 studies), or ineffective (4 studies)	Interventions that detected inappropriate use of PPIs and focused on interpreting the data with the close participation of physicians and patients were more successful.
Boghossian *et al*., 2017 (5), Canada	Cochrane systematic review. Six randomized or quasi-randomized trials comparing at least one deprescription modality with a control (patients maintained daily chronic PPI intake ≥ 28 days). Conducted in Canada, Europe and South Africa.	Adults ≥18 years with chronic PPI treatments diagnosed with non-erosive GERD or moderate degrees of esophagitis. Five studies evaluated on-demand deprescription, one evaluated abrupt deprescription.	In the on-demand deprescription studies, 16.3% had inadequate control of symptoms compared to 9.2% of those who maintained chronic daily PPI use (*P* < 0.0001). Lack of symptom control in 67.9% with abrupt deprescription, versus 22.4% with continuous prescription,	Low quality of evidence favouring continuous use (RR 1.75 95% CI 1.31–2.21)
Walsh *et al*., 2016 (June) (7) Canada	Prospective non-randomized descriptive interventional study conducted in a primary care clinic located in the western area of Toronto	Patients ≥18 years who took PPIs >eight weeks. Prescription tool with the indicated doses and the duration of treatment given to each patient. An electronic notice for each family doctor indicating a patient’s next visit susceptible to deprescription. Measurement evaluating patients’ medical records ten weeks after	43/639 patients with an assigned next appointment comply with inclusion criteria (6,7 %). 11/43 started deprescribing, 6/11 stopped (55%), 4/11 switched to alternative treatment (36%), and 1/11 returned to previous treatment (9%)	16/43 (37%) were no longer taking PPI even though they were on their medication list. The study did not have a control group
Thompson *et al*., 2019 (21), Canada	Before-after study developed in two health centres and a geriatric outpatient clinic in Ontario, Ottawa.	Patients ≥18 years using PPIs >four weeks asymptomatic or did not have an indication to continue. With a questionnaire measured patients’ opinions on deprescription before and after interviewing a pharmacist for 15 min. The pharmacist developed a plan for the patient supervised by the doctor. A telephone follow-up eight weeks after the interview.	12/338 potential participants gave their consent. 75% (7/10 -2 losses-) had reduced the use of PPIs; 5/7 had reduced the dose, and 2/7 had switched to an on-demand-only regimen.	No significant difference in the proportion of patients who changed their minds. The study did not have a control group.
Coyle *et al*., 2019 (22), UK	Prospective non-randomized interventional study, conducted in 26 clinics in the UK in England, Scotland and Wales.	Patients 18–90 years >two consecutive months of active PPI prescription. Pre-selected by specialized nurse and supervised by the family doctors. 20-min appointment with a trained nurse instructed on reducing or stopping PPIs, including using alginate as rescue therapy. Reviewed the patients’ history 12 months after.	4,691/6,249 (75.1%) succeeded in reducing PPI; 541(8.7%) returned to the previous dose. Three centres in England continued the study 24 months after; 64% reduced PPIs, 14.2% was the failure rate.	Considering the increase in the alginate prescription, the net saving obtained at 12 months was 31 716 pounds/year (€ 37 107/year). The study did not have a control group.
Odenthal *et al*., 2020 (23), USA	Prospective non-randomized interventional study, developed in a primary care centre in St Paul, Minnesota.	Patients ≥18 years who took PPIs >eight weeks for GERD without esophagitis or an unknown indication with a scheduled appointment. Interview with a pharmacist during their visit, instructed in a deprescription protocol divided into phases. Interview eight weeks after.	In 26/126 (21%) was possible to start deprescription. 22/26 completed the protocol.19/22 (86%) had a complete abandonment of PPIs, 2/22 (9%) reduced them and 1/22 (5%) took again the PPI starting dose.	7/19 (37%) who discontinued acknowledged that did not need to reduce the doses as explained in the protocol. The study did not have a control group.
Nallapeta *et al*., 2020 (24), USA	Prospective non-randomized interventional study, developed in a primary care-internal medicine clinic in Erie County, Buffalo, New York	Patients ≥50 years seen at least once in the previous twelve months and taking PPIs. Weekly teaching sessions to the doctors on the appropriate PPI prescription/verbal and written deprescribing strategies to the patients. Evaluated rebound symptoms in the patients and monitored prescription monthly per one year	180/201 (90%) did not have a valid indication. The average rate of discontinuation was 51% (92/180) (30% inappropriate chronic use from a baseline of 80% within 12 months). The mean of the discontinuation rate in the pre-study (one-year baseline period) was 2%, in the one-year study period was 32% and in the poststudy period (six months) was sustainable at 50%	Estimated annual savings attributed to deprescription was 13 992 US dollars. The study did not have a control group.
Ayoub *et al*., 2021 (25), USA	Prospective non-randomized interventional study developed in a primary care centre in Oregon	Patients ≥18 years with an active PPI prescription in their therapeutic plan, with an unclear indication or an inappropriate duration. De-escalation protocol explained by a pharmacist in 15-min interview. Follow-up with an interview every two weeks for eight weeks. Final evaluation using a questionnaire, four weeks after the de-escalation had ended.	234/985 were candidates for deprescription. 36/234 could be studied. 15/36 (42%) completed deprescription successfully (no symptoms that would alter daily activities four weeks after)	33% of the patients in the sample had no indication for PPIs. The study did not have a control group.

In the Australian systematic review (Wilsdon *et al.*, 2017), the researchers noted that the published evidence was of low methodological quality, translating into a low level of evidence. They affirm that the uneven results of this review could be explained by the different intervention’s ability to convince the clinician of the need to deprescribe. Based on the review, they established a series of suggestions for any intervention on deprescribing: 1) convincing clinicians of the importance and need to deprescribe by providing effective motivation for it; 2) use control groups; 3) correctly identify the inappropriate prescription; 4) explain the prescription method; 5) explain the severe side effects of the indefinite prescription; 6) carry a prolonged follow-up after the intervention (≥ 24 months). They suggest that deprescription may be more successful with a dose reduction strategy.

Although, according to this review, there are more successful deprescribing strategies than others, the translation of PPI deprescription into good clinical outcomes has not yet been clarified.

In the Canadian Cochrane systematic review (Boghossian *et al.*, 2017), the researchers indicate that, although the risk of symptom reappearance was higher in on-demand therapy than in continuous treatment, most patients tolerated the intervention. Three studies on on-demand deprescription showed a statistically significant reduction in drug consumption (*P* < 0.0001) of 3.8 pills/week (95% CI −4.73 to −2.84), favouring deprescription with moderate quality of evidence. Participant satisfaction was measured by their desire not to continue treatment and inadequate symptom relief. The data in this regard favoured the chronic use of PPIs, although with a low quality of evidence. The investigators note that three studies showed statistical significance in favour of deprescription (*P* < 0.002). Finally, the authors state that there are insufficient data to draw long-term conclusions, given that five studies had a duration of five months and one of 13 weeks.

Walsh et al. (Walsh *et al.*, 2016) developed a prescription tool that consisted of a document based on current gastroenterology guidelines (Canadian and North American) on managing endoscopy-negative GERD. The main barriers detected by the researchers were the refusal of the patients and the lack of time on the part of the doctors. During the project, the number of patients without a PPI indication went from 12 to 4 at the end of the study.

In the study by Thompson et al. (Thompson *et al.*, 2019) even though the researchers found no significant difference in the proportion of patients who changed their minds after the intervention, the interview improved the patients’ expectations, knowledge and confidence in the decision.

Coyle et al. (Coyle *et al.*, 2019) conducted the study according to the National Institute for Health Care Excellence (NICE) guide. They used the electronic medical records of each health centre, and the responsible family doctors had to rule out those pathologies in which the continuous use of PPIs was indicated. The researchers mentioned that no adverse effects were recorded at any point in the study.

In these phases of the protocol developed by Odenthal et al. (Odenthal *et al*., 2020), the medication would be reduced every two weeks and replaced by antiH_2_ until it was suspended, being able to add the use of calcium carbonate gum as rescue medication at any time. Researchers considered deprescription successful if the PPI discontinues and the daily or weekly dose is reduced.

Nallapeta et al. (Nallapeta, Reynolds and Bakhai, 2020) used the American Gastroenterological Association (AGA) guidelines to identify those patients who were taking PPIs inappropriately. The registry used in this study was the electronic medical record. After weekly teaching sessions with the doctors, the researchers conducted subsequent evaluations using tests. Also, pocket guides for physicians developed on the proper management of dyspepsia and side effects caused by long-term use of PPIs.

The de-escalation protocol developed by Ayoub et al. (Ayoub *et al.*, 2021) considered any frequency reduction or discontinuation of the dose, including using antiH_2_ as rescue medication. The NICE guide for GERD was considered to manage PPIs properly.

## Discussion

The most important limitation of this review is the possibility of selection and publication biases. Beyond the high methodological heterogeneity of the studies analysed, some conclusions can be drawn.

In the first place, the inappropriate use of these drugs appears to be very high worldwide, in line with what has been indicated by the scientific literature. These seem to justify establishing effective and straightforward deprescription strategies based on the best available scientific knowledge. The different success rates obtained by the various studies analysed in this review may be due to the different methodologies used when establishing the protocols, sample selection and monitoring of the results. Still, we can conclude that the two factors related to the most successful strategies were a) the clarity and simplicity of the de-escalation protocols, in which patients were instructed on the measures to follow in the event of the reappearance of symptoms, and b) the training of the physicians responsible for deprescribing. Although long-term conclusions cannot be drawn about the effectiveness of these protocols, given that the studies are limited in time (the longest is 24 months), it seems sustainable, despite a reduction of effectivity over time. Longer-term studies would be necessary to confirm this trend. Other barriers to generalizing the results are the small sample size and the absence of control groups (except in the Canadian systematic review).

The most repeated barrier by the researchers of the evaluated studies was access to patient information. However, medical records of different specialists, hospitals and primary care centres were usually not connected. The so-called *polydoctoring* (Ie *et al.*, 2021) makes it difficult or even impedes access to a complete medical record of patients. This situation underlines the importance of the figure of the family doctor as a central element of health care, integrating the different past or current processes that affect their patients and reflecting it in reliable and up-to-date medical records. It can also be concluded that convincing patients and their doctors are crucial for the deprescribing strategies’ success. These patients seem to be more predisposed to deprescription than could be expected as long as it is their doctor who proposes it (Reeve *et al.*, 2015). Interprofessional teams (doctors, pharmacists, nurses and others) working in a collaborative environment could be a key to the success of deprescription approaches and activities.

Finally, it is essential to note that, due to the limited extension of the samples and the strategies, no study has demonstrated a clinical impact of deprescription, although no adverse effects attributable to it have been observed.
